# Classification and Characteristics of Mesenchymal Stem Cells and Its Potential Therapeutic Mechanisms and Applications against Ischemic Stroke

**DOI:** 10.1155/2021/2602871

**Published:** 2021-11-09

**Authors:** Pian Gong, Wei Zhang, Yan He, Jianfeng Wang, Song Li, Songyu Chen, Qingsong Ye, Mingchang Li

**Affiliations:** ^1^Department of Neurosurgery, Renmin Hospital of Wuhan University, Wuhan, Hubei 430060, China; ^2^Regenerative Medicine Lab, Tianyou Hospital, Wuhan University of Science and Technology, Wuhan, Hubei 430064, China; ^3^Department of Neurosurgery, Shanghai Tenth People's Hospital, Tongji University, Shanghai, China; ^4^Center of Regenerative Medicine, Renmin Hospital of Wuhan University, Wuhan, Hubei 430060, China

## Abstract

Ischemic stroke is a serious cerebral disease that often induces death and long-term disability. As a currently available therapy for recanalization after ischemic stroke, thrombolysis, including intravenous thrombolysis and endovascular therapy, still cannot be applicable to all patients due to the narrow time window. Mesenchymal stem cell (MSC) transplantation therapy, which can trigger neuronal regeneration and repair, has been considered as a significant advance in treatment of ischemic stroke. MSC transplantation therapy has exhibited its potential to improve the neurological function in ischemic stroke. Our review describes the current progress and future perspective of MSC transplantation therapy in ischemic stroke treatment, including cell types, transplantation approaches, therapeutic mechanisms, and preliminary clinical trials of MSC transplantation, for providing us an update role of MSC transplantation in ischemic stroke treatment.

## 1. Introduction

Stroke is the third leading cause of death and disability worldwide that brings a huge burden to the healthcare system [1]. One in six people will suffer from stroke in their lifetime, with over 13.7 million occurring strokes every year and causing 5.8 million people deaths [2]. The major type of stroke is the ischemic stroke, which approximately accounts for 70 percent of all strokes [2]. Although advanced methods for ischemic stroke treatment have been dug up in recent years, no therapy can efficiently improve the overall prognosis of patients [3]. Intravenous administration of recombinant tissue plasminogen activator (rt-PA) thrombolysis is the only drug approved by the Food and Drug Administration to treat the acute ischemic stroke within 4.5 hours [4–6]; however, limited by its therapeutic window, less than 5% patients benefited [7]. Mechanical thrombectomy, as the alternative treatment for ischemic stroke, entails an intra-arterial (IA) catheter or stent to remove the occluding thrombus, has been proven to be effective within 6 hours of the onset [8]. Both treatments are so highly time-dependent; thus, new treatment strategy is imminent. As a new method to treat ischemic stroke, stem cell transplantation was proved to be a hopeful treatment by a growing body of animal experiments and a few successful clinical trials [9, 10]. As the most studied subtype of stem cells, mesenchymal stem cells (MSCs) have been regarded as a promising therapeutic option for ischemic stroke [11]. Therefore, this review is aimed at summarizing the current progress as well as the future perspective of MSC transplantation in ischemic stroke treatment.

## 2. Classification and Characteristics of MSCs

**Figure 1:**
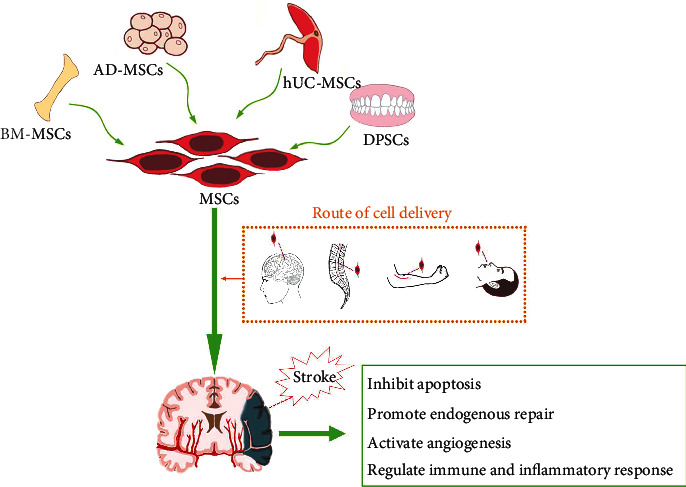
Application of MSCs for ischemic stroke. MSCs can be isolated from the bone marrow, adipose tissue, placenta, and teeth and transplanted to the ischemic brain via intracerebral transplantation, intrathecal administration, intravascular administration, and intranasal administration. MSCs can provide neuroprotection and clinical benefits by inhibiting apoptosis, promoting endogenous repair and angiogenesis, and regulating immune and inflammatory response.

MSCs, also known as mesenchymal stromal cells with properties of self-renewal and multipotential for differentiation, can be isolated from various tissues. As shown in Figure 1, varying from cell sources, MSCs can be obtained from the bone marrow, umbilical cord, adipose tissue, placenta, and tissues that originated from the neural crest and others. Furthermore, they can be differed from other stem cells like the hematopoietic stem cells according to the surface molecules expressed by themselves. MSCs express surface molecules such as CD73, CD90, and CD105, and they do not express the surface molecules like CD34, CD45, HLA-antigen D related, CD14 or CD11b, CD79a, or CD19 [12]. MSCs possess multipotential differentiation ability that they can be differentiated into cells like osteoblasts, chondrocytes, and adipocytes. Moreover, they are easy to be isolated and amplified with low immunogenicity and trophic properties [13, 14]. Unlike embryonic stem cells, the collection, research, and usage of MSCs seldomly raise ethical concerns. Therefore, it is possible to transplanting MSCs to repair multiple injuries.

In the past, researchers obtained MSCs mainly from the bone marrow. MSCs were first found in the bone marrow in 1976 and described as fibroblast-like cells [15]. Later studies revealed that this kind of cells could differentiate into osteogenic, chondrogenic, and adipogenic mesenchymal cell lineages in vitro [16]. Up to date, MSCs in the bone marrow, also known as bone marrow mesenchymal stem cells (BM-MSCs), are the most studied and best characterized MSCs. Currently, the most frequent way to obtain BM-MSCs is bone marrow aspiration accompanied by invasion, pain, and the risk of viral and bacterial contamination [17], of which the quality of the obtained cells is determined by age and physical condition of donors [18]. Despite a very low yield, the amplification ability of BM-MSCs is very strong. In the exponential growth period, its doubling time is about 30 to 33 hours. It is reported that human BM-MSCs can be propagated in vitro for 40 generations and about 100 million times which can still maintain the stemness [16]. A recent investigation suggested that, in rats underwent acute ischemic stroke, intravenous- (IV-) injected human BM-MSCs can survive and migrate along boundary zones adjacent to the ischemic area and differentiate into the neurons and astrocytes in the microenvironment of the ischemic lesion area, as a result of reduced infarct volume and improved neurological function [19]. Moreover, it is reported that transplanting human BM-MSCs into the infarct area not only stimulates angiogenesis and neurogenesis by secreting multiple cytokines like vascular endothelial growth factor (VEGF), basic fibroblast growth factor (bFGF), and TIMP-3 but also induces differentiation of endogenous stem cells, which results in neuroprotection against ischemic stroke [20, 21]. In addition, transplanted human BM-MSCs were shown to inhibit inflammation and neuronal apoptosis in the ischemic brain of rats [22]. The underlying molecular mechanisms of protective effects induced by BM-MSCs are complex and not entirely clear. Interestingly, a study indicated that, shortly after ischemic stroke in mice, the cell proliferation of BM-MSCs was triggered and promoted, leading to the production of downstream myeloid progenitors and increased presence of inflammatory monocytes and neutrophils, suggesting that BM-MSC could be activated by ischemic injury and that the ischemic injury influenced the primary site of hematopoiesis besides the local inflammation in the ischemic brain [23].

Human umbilical cord mesenchymal stem cells (hUC-MSCs) are isolated from the umbilical cord, which are featured with the surface molecules of CD29, CD44, CD51, CD105, SH2, and SH3 except CD34 and CD45 [24]. Lots of research explored the effectiveness of hUC-MSCs in ischemic stroke. It was reported that rats underwent middle cerebral artery occlusion (MCAO) for 2 hours and treated with intracerebral hUC-MSC transplantation 1 d after MCAO operation showed enhancement in neurogenesis and angiogenesis, as a consequence of reduced neurological functional deficits and infarct volume; besides, the hUC-MSCs could be detected for at least 5 weeks in the damaged area [25]. The transplanted hUC-MSCs could differentiate into neural progenitors and cells [26], promote the proliferation of neural stem cells and neural differentiation, produce multiple neurotrophic factors, and prevent inflammatory reaction through regulating the activity of the spleen [27], followed by promoted neurological recovery and reduced mortality in animals underwent ischemic stroke. However, hUC-MSC therapy in ischemic stroke is currently limited due to the risk of infection and tumorigenesis [28]. In conclusion, as a kind of MSC that possesses the unique advantage of low immunogenicity without ethical controversy, hUC-MSC is proposed as an excellent candidate in cell therapy for ischemic stroke.

The placenta is thought to be an abundant source that contains two kinds of MSCs: amniotic mesenchymal stromal cells (hA-MSCs) and chorionic mesenchymal stromal cells (hC-MSCs). Both types of cells can be isolated directly from the placenta at the end of gestation through chorionic villus sampling during an invasive prenatal diagnosis. The characteristics of hA-MSCs and hC-MSCs, including low immunogenicity and powerful proliferation [29, 30], enable both of them various promising biological properties and make it possible to culture them under good manufacturing practice grade, as a result of numbers of clinical trials on placenta-derived stem cell therapy for ischemic stroke [31].

Adipose tissue-derived mesenchymal stem cells (AD-MSCs) are abundant, accessible, and easy to obtain using lip aspiration techniques [32]. Efficacy and safety of human AD-MSC in the treatment of stroke has been confirmed in animal models [33]. AD-MSC transplantation was shown to attenuate the neuronal apoptosis and death to exert significant neuroprotective effects through inhibiting the action of KDM6B/BMP2/BMF axis in rats underwent the MCAO [34].

Dental pulp stem cells (DPSCs), originated from the embryonic neural crest and oral-derived epithelial stem cells, are considered as a kind of autologously applicable cells [35]. A report in 2000 first clarified that DPSCs could be isolated and characterized from the third molar [36]. It is currently well known that DPSCs are easily extracted and obtained from human teeth such as deciduous teeth, impacted third molars, and orthodontically extracted premolars. DPSCs have the MSC-like characteristics of high growth capacity and multilineage differentiation potential that they can convert into multiple kinds of cells like neural cells, chondroblasts, and endothelium formative cells. For the advantages of DPSCs, the major one is the easy accessibility without invasive surgical procedures or ethical concerns; another one is that they can maintain their stem cell characteristics after long-term cryopreservation [37, 38]. Moreover, DPSC culture can also be efficiently established from extracted human molars after cryopreservation for up to one month [39].

In addition, DPSCs showed a superior potential for neurogenic differentiation compared with MSCs from other sources like BM-MSCs and AD-MSCs [40]. Upon induction under neuronal differentiation conditions, DPSCs can differentiate into functionally active neuronal cells like mature neurons, dopaminergic-like cells, Schwann cells, and oligodendrocytes [41, 42]. Even without preinduction of neuronal differentiation, DPSCs can express neural stem cell-like markers like nestin and *β*-III tubulin [40]. Studies showed that DPSCs can express neurotrophic factors like brain-derived neurotrophic factor (BDNF) and VEGF which have been proven to exert neuroprotection against ischemic stroke in both in vitro and in vivo experiments [40]. DPSC-treated primary cortical neurons and astrocytes underwent the oxygen/glucose deprivation (OGD) exhibited promoted neurite regeneration and angiogenesis, and relieved inflammation [19]. DPSC secretion/exosome-implanted rats with ischemic stroke showed promoted nerve cell proliferation, reduced infarct volume and brain edema, and attenuated neurological dysfunction [43, 44]. Moreover, the culture supernatant of DPSCs that is called dental pulp conditioned medium (DPCM) has been reported to contain cytoprotective factor, revascularization factor, and fibrosis inhibitory factor that contribute to neuronal survival, proliferation, and differentiation [45]; importantly, all of which are well known to be the key mechanisms of neuroprotection against cerebral ischemic stroke. Above evidence shows that both DPSC transplantation and DPCM exert therapeutic effects against ischemic stroke. Furthermore, it was reported that no immune rejection was observed in the brain of mice that had been administered with DPSC implantation derived from rhesus monkeys [46], suggesting that the degree of transplantation rejection of DPSCs was low. When combining with neurotrophic factors, DPSCs can repair both the central nerve and the peripheral nerve once they were attacked by various injuries [47–51]. However, few in-depth studies have examined the effects of DPSCs on the ischemic stroke. Therefore, further research is needed and worthy.

## 3. Route of Cell Delivery

To date, MSCs are delivered via intracerebral transplantation, intrathecal administration, intravascular administration, and intranasal administration to repair ischemic-damaged brain tissue, as shown in Figure 1. Despite intracerebral transplantation and intravascular administration are the commonly used methods [52], there is no optimal route of delivery as every method possesses its own vantages and limitations.

Intracerebral transplantation, also known as stereotactic transplantation, directly injects the MSCs into brain parenchyma or cerebrospinal fluid by stereotactic apparatus. When injected via parenchyma, in order to provide a good microenvironment for stem cells to promote graft survival, delivering the MSCs into penumbra or the hemisphere contralateral to the infarct is suggestive [53, 54]. However, intracerebral transplantation may cause mechanical damage and the number of MSCs is limited. Research has showed that not only the endogenous neural stem cells but also exogenous transplanted MSCs are able to migrate to the ischemic region [55, 56]. Yet, other researchers argue that even the stem cells successfully arrived at the center of ischemic area, and some survived the initial ischemic damage, the ideal regenerative niches might only appear several days after the stroke in adult mouse brain [57]. Intracerebroventricular injection delivers the MSCs in cerebrospinal fluid mainly to treat brain functional diseases, especially in cerebral ischemia. It was verified that implanted hUC-MSCs by intracerebroventricular injection migrated into the periventricular tissue, followed by promoted functional recovery in the rat model of hypoxic-ischemic encephalopathy [58]. In terms of the timing of administration, both early (12 hours after stroke) and delayed (7 days after stroke) administrations have been proved effective in improving functional outcomes of rats underwent ischemic stroke [59, 60]. Owing to the importance to control the increased intracranial pressure, multiple administrations of intracerebral transplantation are thought to be impractical, especially for the patients in critical conditions [61]. Intracerebral injection bypasses the blood-brain barrier (BBB) and allows more MSCs into the ischemic lesion. However, as an invasive operation, it is highly technique-sensitive and equipment-dependent [62]. For instance, the positioning accuracy of injection site on MSC transplantation can reach 0.1 mm using a stereo orientation technique [63].

Intrathecal injection delivers MSCs throughout the entire neuraxis without the invasive brain surgery, making it different from the intraparenchymal and intracerebroventricular administrations [64]. Lim et al. found that therapeutic effects of intrathecal injection of hUC-MSCs can be achieved at a lower dosage in treating cerebral ischemic stroke of rats, compared with intravenous administration [65]. A prospective phase II trial has been initiated in 2019 to assess the effectiveness of allogenic BM-MSC transplantation in severe ischemic stroke, in which eligible patients received BM-MSCs intrathecally at the subacute phase (30 to 90 days following onset) and follow-up assessment were conducted at 7, 30, 90, 180, and 360 days after the injection; after all, the project is in progress with no conclusions published so far [66]. This study may provide a good knowledge of intrathecally implanted BM-MSC therapy for severe ischemic stroke.

Intravascular injection is a safe and feasible delivery way of MSCs, including IV and IA administrations. We find an interesting phenomenon that most clinical trials have used IV injection, and studies with smaller case series preferred IA route [67–69]. Compared with intracerebral injection, intravascular injection is less invasive and allows higher dose and bigger volume of MSCs.

Delivered via IV route, cells are expected to pass the BBB to reach the infarct site of brain and function properly to regenerate new nerve tissue. A research was conducted that the neural stem cells were transplanted into mice at 24 h after ischemic stroke through IA and IV methods; the results of which showed that IV route leaved the cells traveling through the systemic and pulmonary circulations where cells were more likely to be entrapped in other organs like the spleen, liver, and lungs that 94% of cells were detected in the lungs at 1 week after stroke, resulting in only a small part of injected portion can reach the brain, and that IA route leaved 69% of MSCs in the brain several hours after injection and 93% at 7 days [70]. It was reported that, in rats suffering ischemic stroke, at 14 days after BM-MSC transplantation, the implanted cells through IV injection could not be detected in the ischemic brain and most of them were trapped in the lung, and 35% of intracerebrally injected MSCs migrated along the corpus callosum to the ischemic region [21]. In the MCAO model of rats, AD-MSCs were injected via IA and IV routes at 24 h after onset and results showed that at 1-7 days after implantation, the expression level of neurotrophic substance, such as BDNF and VEGF, was increased and level of proapoptotic factors like caspase-3 and TNF-*α* was decreased via IA route compared with the IV delivery [71]. IA delivery seems to circumvent the systemic circulation [52], via which the cell number entering the brain was 5 times higher than IV [72]. However, IA injection takes the MSCs nearly 24 hours up to 10 days to reach the brain parenchyma [73, 74].

The intranasal route is another less invasive therapeutic option. By passing the BBB, BM-MSCs that were transplanted 24 h after ischemic stroke in mice could reach the ischemic cortex as early as 1.5 hours postnasal administration, which deposit outside the blood vessels [75]. Cell tracking techniques indicated that the cells can enter the olfactory sheath through the extension adjacent to the olfactory filament after passing through the sieve plate or moving along the surface of the cortex into cerebrospinal fluid and then go into the brain parenchyma [76, 77]. This route minimizes, if not eliminates, the cell dispersion in systemic circulation to peripheral organs such as the lung. A study showed that the 9-day-old mice administered with MSCs intranasally at 10 days after hypoxic-ischemic brain damage suggested that both of the somatosensory cortex and hippocampus were dramatically regenerated and glial scar around the ischemic site was eliminated at 18 days after the cell therapy [78]. Another study implied that 10-day-old rats administered with hUC-MSCs intranasally at 24 h posthypoxic-ischemic displayed relieved neuroinflammation and promoted neural regeneration [79].

## 4. Treatment Mechanisms of MSCs in Ischemic Stroke

### 4.1. MSCs Regulate Immune and Inflammatory Response

**Figure 2:**
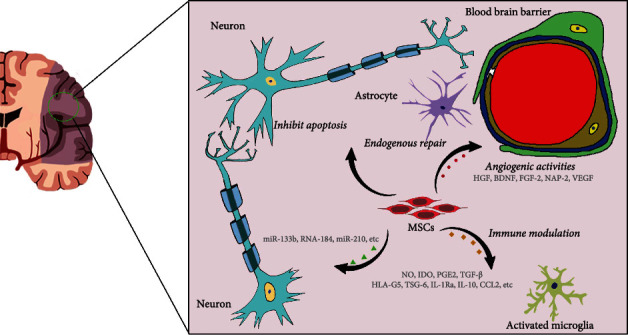
Treatment mechanisms of MSCs in ischemic stroke. MSCs produce NO, IDO, PGE2, TGF-*β*, HLA-G5, TSG-6, IL-1Ra, IL-10, CCL2, etc., to weaken harmful immune and inflammation responses. MSCs induce activation of microglia and persistent reactive astrogliosis. MSCs interact with microRNAs, like miR-133b, RNA-184, and miR-210, to provide neuroprotective functions. MSCs can secrete biologically active cytokines or factors including BDNF, GDNF, NGF, cCSF, SCF, bFGF, PDGFAA, angiopoietin-2, NAP2, and VEGF to promote angiogenic activities and attenuate blood-brain barrier disruption.

Both of immune and inflammatory responses are proven to be significantly involved in the pathogenesis of ischemic stroke. Once the ischemia attacks, the activated innate immunity quickly triggers and promotes the neuroinflammation (Figure 1), as a result of the migration of immune cells from periphery into the ischemic brain [80]. At early stage of ischemic stroke, inflammation limits and relieves the ischemic stress, which is beneficial for the patients. But the following uncontrolled inflammation induced by immune cells, for example, the neutrophils, macrophages, NK cells, and T cells, aggravates the ischemic injury [81, 82]. The ischemic injury is a result of the BBB breakdown, the expression of harmful molecules produced by neural cells, production of glia cell activation-derived proinflammatory factors, and the leukocyte accumulation. MSCs are proven to produce immune regulatory factors such as NO (in mice), IDO (in human), PGE2, TGF-*β*, HLA-G5, TSG-6, IL-1Ra, IL-10, and antagonistic variants of CCL2 that weaken harmful immune and inflammation responses and promote tissue repair and regeneration (Figure 2) [83, 84]. The soluble factors derived from MSCs are contributed to the suppression of T cell proliferation, and the TGF-*β* secreted by MSCs prevents the production of PGE2 and HO-1 and also inhibits the autocrine proliferation of IL-2-dependent T cells (Figure 2). In cerebral ischemia, hUC-MSC injection through the tail vein has been shown to modulate TGF-*β*, leading to the conversion of naïve CD4^+^ T cells into Th17/Treg and regulation of peripheral immune response, followed by inhibited neuroinflammation and attenuated ischemic injury [85]. In addition, MSCs utilize their property on cell cycle arrest to suppress the IFN-*γ*^+^/CD8^+^ T cell proliferation through modulating the expression of cyclin D2 and p27kip1, both of which deeply influence the cell cycle of T cells [86]. Moreover, MSC-derived doleamine 2,3-dioxygenase (IDO), PGE2, and TGF-*β*1 can downregulate the expression of activated receptors (NKp30, NKp44, and NKG2D), reduce cytotoxicity, inhibit the production of inflammatory cytokines (IFN-*γ* and TNF-*α*), and inhibit IL cytotoxic T cell and NK cell proliferation (Figure 2) [87, 88]. Early MSC transplantation significantly drives the IL-10 expression, following the decrease in TNF-*α* production in the ischemic area. BM-MSC transplantations have important roles in attenuating neutrophil infiltration, astrocyte apoptosis, MMP-9 activation, and aquaporin-4 (AQP4) upregulation through suppressing intracellular adhesion molecule 1 (ICAM-1) and activating the p38 signaling pathway, leading to accelerated and enhanced glial scar formation, and reduced BBB disruption and ischemic lesion volume (Figure 2) [89–91].

Splenic inhibition may be another mechanism of MSCs to lighten the immune inflammation. BM-MSC transplantation allows a potential approach for BBB protection in cerebral ischemia that the cell-based therapy attenuates the adverse effect induced by the spleen which increases the BBB permeability and aggravates the BBB disruption [92, 93]. Besides, BM-MSC therapy modulates the peripheral immune response (Figure 1), which is mainly triggered and promoted by the spleen through releasing lymphocytes and proinflammatory factors into the circulatory system at early stage of ischemia [27, 94, 95].

### 4.2. MSCs Provide Neurotrophic Functions

At present, the most widely accepted mechanism how MSCs exert the protective effects in ischemic stroke is the neurotrophic factor produced by MSCs through endocrine or paracrine pathway [96]. It has been clarified that the MSCs produce various biologically active cytokines or growth factors like BDNF and bFGF that are crucial for neural regeneration, white matter remodeling, and synaptic plasticity [97–99]. For example, studies have shown that BDNF and bFGF expressed by MSCs inhibit the neural death and apoptosis partly through interacting with tyrosine kinase receptors in the animal model of ischemic stroke (Figure 2). As a result, MSC transplantation significantly increased the number of multiple nerve cells synaptophysin, as well as synaptic density, number of myelinated axons, and protuberance growth in the ischemic border area [100]. The increased intensities of oligodendrocyte progenitor cells and mature oligodendrocytes are observed in the border zone of lesion due to BM-MSC transplantation [59]. Moreover, BM-MSC transplantation enhances white matter remodeling through activation of microglia and persistent reactive astrogliosis (Figure 2), leading to the better long-term neurological outcomes [59, 101].

MicroRNAs are strongly linked to MSCs, which play a neuroprotective role in ischemic stroke. MicroRNA profiling analysis revealed that many microRNAs were significantly changed after ischemic stroke [102, 103]. Xin et al. found that the exosomes from MSCs can modulate the interaction between various microRNAs and neural cells to the structural and functional recovery of neural cells in cerebral ischemia (Figure 2) [104, 105]. It has been proposed that MSCs comodified by targeted peptide and miR-133b can be used as potential therapeutic drugs for cerebral ischemia (Figure 2) [106]. In addition, MSCs act on extracellular vesicles (EVs) in region site to mitigate ischemic injury that the interaction between them promotes neurogenesis and angiogenesis [107]. There is no doubt that MSC treatment has a potential therapeutic value in ischemic stroke with the ability of opening up new avenues and strategies.

### 4.3. MSCs Induce Angiogenic Activities

Angiogenesis is highly related to the functional recovery of ischemic stroke (Figure 1). In response to the attack of the ischemic stroke, the vascular endothelial cells in the CNS exhibit strong proliferation capability to supply the injured tissue with more oxygen and nutrition. In the next few months after the stroke, as vascular remodeling and vascular density increasing, neuroblasts gradually migrate to the damaged brain area to repair the injured tissue. Existing evidence suggested that MSCs can acquire angiogenic properties through paracrine or autocrine production of appropriate cytokines [108, 109]. Implanted BM-MSCs release many angiogenic growth factors and neurotrophic factors like angiogenin, hepatocyte growth factor, BDNF, and fibroblast growth factor-2 (FGF-2), insulin-like growth factor-1, neutrophil activating protein-2 (NAP-2), and VEGF (Figure 2) [109]. In addition, some researchers have found that, in the area around the infarction, BM-MSCs facilitate the production of various neuroprotective factors, including stromal cell-derived factor-1 (SDF-1), BDNF, platelet-derived growth factor AA (PDGF-AA), basic fibroblast cell growth factor, angiopoietin-2, CXC chemokine ligand 16, NAP-2, and VEGF receptor-3 (Figure 2) [110]. In rats suffering ischemic stroke, BM-MSC treatment started at 24 h after onset markedly increases the microvessel density, as a consequence of enhanced angiogenesis in the boundary zone [101, 111]. Zacharek et al. found that coculture of astrocytes with MSCs increased the expression of VEGF and Ang1/Tie2 and significantly increased capillary-like formation of mouse brain endothelial cells [112], resulting in the promotion of the angiogenesis to accelerate tissue repair (Figure 1).

## 5. Clinical Trials

Although preclinical data are promising in terms of both safety and therapeutic efficacy, clinical verification is inevitable for MSCs to treat patients with cerebral ischemia. Up to now, about 1000 clinical trials focused on MSC therapy are currently registered on ClinicalTrials.gov and 20 clinical trials (Table 1) focus on the therapeutic effects of MSCs for cerebral ischemia, among which 4 have been completed and 3 have been withdrawn. A phase II clinical trial from China, in which 10 participants suffering acute ischemic stroke were treated with AD-MSCs via the IV route within 2 weeks after the onset, concluded that AD-MSC implantation was safe and efficient and could improve the neurological function of patients with severe stroke at two years after ischemic stroke [113]. Another 4-year open trial in China involved 18 participants with acute cerebral ischemic stroke showed that participants in the MSC-treated group had fewer serious adverse events compared with the vehicle group and concluded the long-term safety of MSC treatment for acute cerebral ischemic stroke [114]. In a randomized controlled trial with a 2-year follow-up, 16 patients received the MSC transplantation through the IV route showed improved motor recovery through sensorimotor neuroplasticity, suggesting that MSC treatment was safe and feasible for ischemic stroke [67].

Moreover, clinical trials of 36 patients from Levy et al. [115] showed that the proportion of patients that treated with allogeneic BM-MSCs (1.6 × 10^6^/kg) via IV route with good functional recovery (Barthel score ≥ 95) increased from 11.4% of the baseline to 27.3% at 6 months and 35.5% at 12 months. Levy et al. concluded that allogeneic BM-MSC injection via IV route was accessible and reasonable in treatment of chronic stroke and suggested behavioral gains in patients with substantial functional defects. In addition, a randomized controlled clinical trial registered as ChiCTR-INR-16008908 focused on intrathecal injection of allogeneic BM-MSC four infusions (1 × 10^6^ cells/kg body weight) once a week at 1 to 3 months after onset of ischemic stroke is still in progress [66].

## 6. Conclusions and Prospects

At present, MSCs are notably available from multiple sources. Furthermore, they are immunotolerant and hold unequivocal postnatal multilineage potential. MSC transplantation is indeed an excellent therapeutic technique to treat ischemic stroke; however, of which the optimal therapeutic protocols, in terms of MSC subtype, number, preparation, and timing, need to be further studied. Although preclinical studies have shown that MSC therapy occupies with safety and efficacy in the treatment of ischemic stroke, some investigators concern that MSC transplantation may lead to tumor growth, immunodepression, and adverse events of the respiratory system, particularly the pulmonary embolism [63]. In addition to the encouraging phase I and phase II data, large-scale phase III clinical trials are required to clear the aforementioned doubts. Hence, we should further conduct not only preclinical studies but also clinical researches to illustrate the effectiveness of MSCs in cerebral ischemia treatment.

## Figures and Tables

**Table 1:** Summary of clinical trials on mesenchymal stem cells and ischemic stroke.

S. no.	Study title	Status	Phase	Cell type	Patient sample size	Route of therapy	Treatment time	Region	Outcomes
1	Umbilical cord-derived mesenchymal stem cells for ischemic stroke	Recruiting	II	hUC-MSCs	200	IV	Within 6 months after onset	Shenyang, China	No results available
2	Safety of escalating doses of intravenous bone marrow-derived mesenchymal stem cells in patients with a new ischemic stroke	Withdrawn	I/II	BM-MSCs	0	IV	Within 24-72 hours after onset	California, United States	No results available
3	Reparative therapy in acute ischemic stroke with allogenic mesenchymal stem cells from adipose tissue, safety assessment, a randomized, double-blind placebo controlled single-center pilot clinical trial	Completed	II	AD-MSCs	19	IV	Acute cerebral infarction of less than 12 h from stroke onset	Madrid, Spain	Safe, feasible, and has improved neurologic recovery in patients with severe stroke
4	Allogeneic adipose tissue-derived mesenchymal stem cells in ischemic stroke	Recruiting	II	AD-MSCs	30	IV	The first 4 days (+/-1) from acute stroke symptoms onset	Madrid, Spain	No results available
5	Combination of conditioned medium and umbilical cord-mesenchymal stem cell therapy for subacute stroke infarct	Not yet recruiting	I/II	hUC-MSCs	15		Acute or subacute phase	Jakarta, Indonesia	No results available
6	Mesenchymal stem cells for the treatment of acute ischemic stroke	Recruiting	I	UMC119-06	9	IV	Within 48 to 168 hours after onset	Taiwan	No results available
7	Mesenchymal stromal cells for ischemic stroke	Withdrawn	I/II	BM-MSCs	0	IV	3-10 days after stroke	Houston	No results available
8	Autologous bone marrow mesenchymal stem cell transplantation for chronic ischemic stroke	Unknown	I	BM-MSCs	40	IV	With stroke history of more than 6 months, less than 60 months	Guangdong, China	No results available
9	Allogenic mesenchymal stem cell-derived exosome in patients with acute ischemic stroke	Recruiting	I/II	MSC-derived exosome	5	IV	Acute phase within 24 h after onset	Tehran, Iran	No results available
10	Evaluate the safety and explore efficacy of umbilical cord mesenchymal stem cells in acute ischemic stroke	Recruiting	I	hUC-MSCs	14	IA and IV	Acute phase	Taiwan	No results available
11	Allogeneic mesenchymal stem cells for the survivors of ischemic stroke trial (ASSIST)	Not yet recruiting	I/IIa	it-hMSCs	60	IV	More than 6 months after onset	Beijing, China	No results available
12	Perinatal arterial stroke treated with stromal cells intranasally	Recruiting	I/II	BM-MSCs	10	Nasal route	Within the first week after onset	Utrecht, Netherlands	No results available
13	Clinical plan of ischemic stroke	Recruiting	I	it-hMSCs	60	IV	More than 6 months after onset	Beijing, China	No results available
14	Intravenous stem cells after ischemic stroke	Completed	II	BM-MSCs	31	IV	Within 6 weeks after onset	Grenoble, France	Safe, feasible, and improved motor recovery
15	Umbilical cord derived mesenchymal stem cell treatment in ischemic stroke	Unknown	II	hUC-MSCs	2	IV	Within 3 months after onset	Beijing, China	No results available
16	The STem cell Application Researches and Trials In NeuroloGy-2 (STARTING-2)	Unknown	III	Autologous MSCs	60	IV	Within 90 days after onset	Seoul, Korea	Feasible and safe but not associated with improvements in the 3-month mRS score and 90-day outcomes in patients with chronic stroke
17	Autologous bone marrow stromal cell and endothelial progenitor cell transplantation in ischemic	Completed	I/II	BM-MSCs	20	IV	Within 7 days after the onset	Guangdong, China	No toxicity events or infusional or allergic reactions and may improve recovery after stroke
18	A study of allogeneic mesenchymal bone marrow cells in subjects with ischemic stroke	Completed	I/II	BM-MSCs	38	IV	More than 6 months after the onset	California, United States	Safe and suggested behavioral gains
19	Autologous bone marrow mesenchymal stem cell transplantation for chronic stroke	Unknown	I	BM-MSCs	30	Intracerebral injection	Within 3 to 60 months after the onset	Zhejiang, China	No results available
20	Ex vivo cultured adult allogenic MSCs in ischemic cerebral stroke	Withdrawn	I/II	Allogenic MSCs	0	Intravenous ex vivo cultured	Within 10 days after the onset	Malaysia	No results available

hUC-MSCs: human umbilical cord mesenchymal stem cells; BM-MSCs: bone marrow mesenchymal stem cells; AD-MSCs: adipose tissue-derived mesenchymal stem cells; it-hMSCs: ischemia tolerant human allogeneic bone marrow mesenchymal stem cells; IA: intra-arterial injection; IV: intravenous injection.

## Abbreviations

MSCs: Mesenchymal stem cells
BM-MSCs: Bone marrow mesenchymal stem cells
IV: Intravenous
VEGF: Vascular endothelial growth factor
bFGF: Basic fibroblast growth factor
hUC-MSCs: Human umbilical cord mesenchymal stem cells
MCAO: Middle cerebral artery occlusion
hA-MSCs: Human amniotic mesenchymal stromal cells
hC-MSCs: Human chorionic mesenchymal stromal cells
AD-MSCs: Adipose tissue-derived mesenchymal stem cells
DPSCs: Dental pulp stem cells
BDNF: Brain-derived neurotrophic factor
DPCM: Dental pulp conditioned medium
BBB: Blood-brain barrier
IA: Intra-arterial
NAP-2: Neutrophil activating protein-2.
